# Chromosomal abnormalities and structural defects in fetuses with increased nuchal translucency at a Chinese tertiary medical center

**DOI:** 10.3389/fmed.2023.1158554

**Published:** 2023-05-23

**Authors:** Huijing Zhang, Shuang Wang, Chunli Feng, Hongyan Zhao, Weiwei Zhang, Yu Sun, Huixia Yang

**Affiliations:** ^1^Department of Obstetrics and Gynecology, Peking University First Hospital, Beijing, China; ^2^Department of Ultrasound, Tongliao Second People’s Hospital, Tongliao, Inner Mongolia, China; ^3^Department of Obstetrics and Gynecology, Chengde Central Hospital, Chengde, Hebei, China; ^4^Department of Ultrasound, Aerospace Central Hospital, Beijing, China

**Keywords:** increased NT, cut-off, chromosomal abnormalities, structural defects, Chinese population

## Abstract

**Objectives:**

To explore the pregnancy outcomes of fetuses with increased NT thickness.

**Methods:**

This was a retrospective study of fetuses with increased NT (≥95th centile) at 11–14 weeks of gestation between January 2020 and November 2020.

**Results:**

Among 264 fetuses with increased NT, the median of CRL and NT was 61.2 mm and 2.41 mm. Among them, 132 pregnancy women chose invasive prenatal diagnosis (43 cases of chorionic villus sampling (CVS), 89 cases of amniocentesis). Eventually, 16 cases of chromosomal abnormalities were discovered, including 6 cases (6.4%) of trisomy 21, 4 cases (3%) of trisomy 18, 1 case (0.8%) of 45, XO, 1 case (0.8%) of 47, XXY and 4 cases (3.03%) of CNV abnormalities. The major structural defects included hydrops (6.4%), cardiac defects (3%), and urinary anomalies (2.7%). The incidences of chromosomal abnormalities and structural defects in the NT < 2.5 mm group were 1.3 and 6%, while the incidences were 8.8 and 28.9% in the NT≥2.5 group.

**Conclusion:**

Increased NT was associated with high risk of chromosomal abnormalities and structural anomalies. Chromosomal abnormalities and structural defects could be detected when NT thickness was between 95th centile and 2.5 mm.

## Introduction

In recent decades, fetal nuchal translucency (NT) has become an important part of the combined screening for aneuploidy in early pregnancy (1). NT thickening was also associated with fetal structural abnormalities and genetic syndromes (2–7). In clinical practice, different centers all around the globe choose different cut-off values for aneuploidy screening, mainly including 2.5 mm (8), 3.0 mm (9), and the 95th percentile (10). According to results recently published by our center, when the 95th percentile was used as the cut-off value, the sensitivity of trisomy 21 screening was significantly higher than when the cut-off value was 2.5 mm (72.73% vs. 54.55%). Therefore, as a marker to screen for Down syndrome, our center believes that using the 95th percentile as the cut-off value is more reasonable (11).

However, the distribution of chromosomal abnormalities and structural defects in the increased NT population in our dataset were not known. Therefore, we continued to explore the pregnancy outcomes of fetuses with increased NT thickness.

## Methods

### Study population

We retrospectively collected data from singleton pregnant women who underwent NT scan in the Department of Obstetrics and Gynecology, Peking University First Hospital from 1 January 2020 to 30 November 2020. Among 4,879 cases of singleton pregnancy, there are 288 (5.9%) fetuses, whose NT thickness were at or more than 95th percentile. However, 24 cases were lost to follow-up. Consequently, 264 cases were included for analysis. The study was approved by the Institutional Review Board of Peking University First Hospital [protocol code 2013(572)].

### NT measurement

The sonographers who performed NT ultrasonography were all qualified with more than 5 years of experience in NT scan. The methods measuring fetal crown-rump length (CRL) and NT were performed in strict accordance with the measurement criteria established by Fetal Medicine Foundation (FMF) (12). The requirements to obtain a standard NT measurement were as follows: (1) CRL measurements were from 45 to 84 mm; (2) the median sagittal section of the fetus in the natural state was taken; meanwhile, the nasal bone (three-line sign) and the upper jaw needed to be displayed at the same plane; (3) the image was amplified to the maximum only to show the fetal head and upper chest; (4) the distance between the inner edge of the skin and the surface of the cervical spine was measured; (5) the maximum value of multiple measurements was reported. The cut-off value of NT used in our hospital at that time was 2.5 mm.

### Data collection

The maternal age, the CRL and NT measurements, non-invasive prenatal testing (NIPT) result, prenatal diagnosis (amniocentesis, chorionic villus puncture) results, structural anomalies and fetal outcomes were collected.

### Screening and diagnosis protocol

If a fetus has an NT thickness less than 2.5 mm, CRL < 79.5 mm and no other high-risk factors (advanced maternal age, no history of adverse pregnancy, no history of genetic diseases, etc.), first trimester combined screening were offered. Meantime, the pregnant woman has the right to decide if she wants NIPT or not. On the contrary, if a fetus has an NT thickness ≥ 2.5 or a high risk result of combined screening test/NIPT or other high-risk factors, the pregnant woman could choose invasive procedures on her own. Both karyotype and chromosomal microarray analysis (CMA) would be performed for cases who agreed of chorionic villus sampling (CVS) or amniocentesis.

### Statistical analysis

In this study, IBM SPSS 25.0 software was used for statistical analysis. Measurement data were expressed as the mean (standard deviation) or median (quartile); categorical data were expressed as the number of cases (percentage). The chi-square test (or Fisher’s exact test) was used for comparisons between groups. *p* < 0.05 was considered statistically significant.

### Patient and public involvement

No patients were engaged in setting the research question or the outcome measures, nor were any patients involved in the study’s design or implementation.

## Results

### Clinical characteristics of pregnancies with increased NT thickness

As shown in Table 1, the average age of 264 pregnant women was 32.09 years old. The median of CRL and NT was 61.2 and 2.41 mm. Among them, 132 pregnancy women (50%) chose invasive diagnosis test (43 cases of chorionic villus puncture, 89 cases of amniocentesis). Eventually, 16 cases of chromosomal abnormalities were discovered (Table 1).

**Table 1:** Clinical characteristics of pregnancies with increased NT thickness (≥95th centile).

Characteristic	Value
Maternal age (y)	32.09 (4.27)
CRL at NT scan (mm)	61.2 (56, 65.55)
NT measurement (mm)	2.41 (2.23, 2.96)
Invasive diagnostic test	132 (50%)
CVS	43 (16.3%)
Amniocentesis	89 (33.7%)
Chromosomal abnormalities	16/132 (12.2%)
NIPT	80 (30.3%)
Detection rate for aneuploidy	1/2 (50%)
Complicated with other structural defects or soft markers	42 (15.9)
Hydrops	17 (6.4)
Cardiac defects	8 (3)
VSD	5
AVSD	1
ToF	1
Aortic atresia	1
Urinary anomalies	7 (2.7)
Duplex kidney	3
Hydronephrosis	3
Hypospadias	1
Facial defect	7 (2.7)
Hypoplastic nasal bone	3
Cleft palate	3
Cyclopia	1
Abnormal DV blood flow	6 (2.3)
CNS anomalies	5 (1.9)
Ventriculomegaly	3
Absent corpus callosum	1
Holoprosencephaly	1
Omphalocele	4 (1.5)
Skeletal anomalies	3 (1.1)
Fetal akinesia deformation sequence	1
Short bones	1
Polydactyly	1
GI anomalies	2 (0.7)
Intestinal duplication	1
Absent stomach	1
Outcome of pregnancy	
Live birth	233 (88.3)
IUD	3 (1.1)
T18	1
Hydrops	1
Omphalocele and abnormal DV blood flow	1
TOP	28 (10.6)
Chromosomal abnormalities	12
Structural defects	15
Isolated increased NT (NT = 4.36 mm)	1

Data are given as mean (standard deviation), median (interquartile range) or n (%). CRL, crown-rump length; NT, Nuchal translucency; CVS, chorionic villus sampling; NIPT, non-invasive prenatal testing; VSD, ventricle septum defect; AVSD, atrial-ventricle septum defect; ToF, Tetralogy of Fallot; DV, ductus venous; CNS, central nervous system; GI, gastrointestinal; IUD, intrauterine death; TOP, termination of pregnancy.

When NT thickness was above 95th percentile, the major structural defects found in the anomaly scan included hydrops (17/264, 6.4%), cardiac defects (8/264, 3%), urinary anomalies (7/264, 2.7%), facial defects (7/264, 2.7%), and abnormal DV blood flow (6/264, 2.3%). There were 10 (3.8%) cases involving multiple anomalies.

### Distribution of chromosomal abnormalities in different NT thickness

We further divided the cases into subgroups according to different NT thickness. Apart from 132 pregnant women who declined the invasive diagnosis test, the distribution of chromosomal abnormalities for the rest 132 cases were listed as Table 2. With the increasing NT thickness, a rising tendency of the chromosomal abnormalities proportion was noted with slight fluctuation, ranging from 4.5 to 100%.

**Table 2:** Distribution of chromosomal abnormalities detected with different NT thickness subgroups.

	Invasive diagnosis								No invasive diagnosis tests
NT (mm)	Total	Normal result	Chromosomal abnormalities						
			Total	T21	T18	45, XO	47, XXY	CNVs	
[95th centile-2.5)	34	32 (94.1)	2 (5.9)	1	0	0	1	0	116
[2.5–3.0)	47	44 (93.6)	3 (6.4)	1	0	0	0	2	4
[3.0–3.5)	22	21 (95.5)	1 (4.5)	1	0	0	0	0	1
[3.5–4.5)	15	11 (73.3)	4 (26.7)	2	1	0	0	1	0
[4.5–5.5)	7	4 (57.1)	3 (42.9)	1	1	0	0	1	1
[5.5–6.5)	1	0 (0)	1 (100.0)	0	1	0	0	0	1
≥ 6.5	6	4 (66.7)	2 (33.3)	0	1	1	0	0	9

[value a, value b) meant that value a was included, while value b was excluded. T21, trisomy 21; T 18, trisomy 18; CNVs, copy number variants.

### Distribution of structural defects in different NT thickness

Likewise, when the NT were increasingly thickened, the percentage of hydrops and cardiac defects increased to 86.7 and 100.0%, respectively (Table 3).

**Table 3:** Distribution of structural defects with different NT thickness subgroups.

NT (mm)	Total	Structural defects								
		Hydrops	Cardiac defects	Urinary anomalies	Facial defects	Abnormal DV blood flow	CNS anomalies	Omphalocele	Skeletal anomalies	GI anomalies
[95th centile-2.5)	150	1 (0.7)	1 (0.7)	4 (2.8)	1 (0.7)	0	2 (1.4)	0	0	0
[2.5–3.0)	51	0	1 (2.0)	2 (4.0)	0	1 (2.0)	0	0	0	1 (2.0)
[3.0–3.5)	23	1 (4.3)	0	1 (4.3)	2 (8.6)	0	0	0	1 (4.3)	0
[3.5–4.5)	15	0	1 (6.7)	0	0	0	0	0	0	0
[4.5–5.5)	8	3 (37.5)	2 (25.0)	0	1 (12.5)	1 (12.5)	2 (25.0)	1 (12.5)	0	1 (12.5)
[5.5–6.5)	2	0	2 (100.0)	0	1 (50.0)	0	0	0	1 (50.0)	0
≥ 6.5	15	13 (86.7)	1 (6.7)	0	2 (13.4)	4 (26.8)	1 (6.7)	3 (20.0)	1 (6.7)	0

[value a, value b) meant that value a was included, while value b was excluded. CNS, central nervous system.

### Proportion of chromosomal abnormalities and structural anomalies with different NT cut-off value

As shown in Table 4, the incidences of chromosomal abnormalities and structural defects in the NT < 2.5 mm group were 1.3 and 6%, while the incidences were 8.8 and 28.9% in the NT ≥ 2.5 group. Similar results were displayed with NT cut-off as 3.0 mm and 3.5 mm. When comparing the results of chromosomal abnormalities and structural defects among the NT < 2.5 mm group, NT < 3.0 mm group and NT < 3.5 mm group, their percentage were similar (1.3% vs. 1.5% vs. 1.8, 6.0% vs. 7.0% vs. 8.6%).

**Table 4:** Incidence of aneuploidy and CNV in NT < 3.5 mm and ≥ 3.5 mm subgroups.

Chromosomal abnormalities and structural anomalies	NT cut-off	<2.5	≥2.5	χ^2^	<3.0	≥3.5	χ^2^	<3.5	≥3.5	χ^2^	*P*	*P*	*P*
Chromosomal	Normal	148 (98.7)	101 (88.6)		196 (97.5)	53 (84.1)		216 (97.3)	33 (78.6)		Aneuploidy	2 (1.3)	10 (8.8)	8.569 0.003	3 (1.5)	9 (14.3)	18.237 <0.001	4 (1.8)	8 (19.0)	24.667 <0.001	Pathogenic CNVs	0 (0.0)	3 (2.6)	4.321 0.069	2 (1.0)	1 (1.6)	0.256 0.517	2 (0.9)	1 (2.4)	1.024 0.354
Structural	Normal	141 (94.0)	81 (71.1)		187 (93.0)	35 (55.6)		203 (91.4)	19 (45.2)		Structural defects	9 (6.0)	33 (28.9)	25.496 <0.001	14 (7.0)	28 (44.4)	50.364 <0.001	19 (8.6)	23 (54.8)	56.357 <0.001

### Details of abnormal CNVs

In this study, 4 cases (4/132, 3%) had abnormal CNVs. After genetic counseling, two pregnant women eventually chose to terminate the pregnancy (1 case of suspected pathogenic CNVs, 1 case of pathogenic CNVs with multiple fetal structures) (Table 5).

**Table 5:** Overview of CNVs.

Case	MA (y)	CRL/NT (mm)	Ultrasound findings in the second trimester	CNVs	Related gene	Possible symptoms	Outcome
48	35	64.2/2.76	Normal	Arr[GRCh37]16p13.11(14968855_16251122)X1 1.282 Mb deletion, possible pathologic	NDE1	Growth retardation, seizure, microcephly etc.	TOP
104	28	55.7/3.55	Normal	arr[GRCh37]5q13.2(69238677_70587018)X1 1.384 Mb deletion, VUS			Live birth normal neurobehavioral development
216	30	54.4/4.87	Multiple: VSD, ventriculomegaly	arr[GRCh37]10q23.1q23.3(87333283_89970915)X1 2.637 Mb deletion, GRID1, pathogenic	GRID1	Growth retardation, seizure, multiple anomalies	TOP
405	35	48.3/2.57	Normal	arr[GRCh37]1q21.1(145415190_145799602)X1, 0.384 Mb, deletion, possible pathogenic	RBM8A	Growth retardation, intellectual dysfunction, microcephaly, CHD	Live birth

## Discussion

In our study, the incidence of increased NT thickness was 5.9% (288/4879), which was slightly higher than the incidence reported by Snijders et al. (4.9%, 4767/96127) (10). The reason for this difference may be that our hospital is a tertiary care center. In addition to providing prenatal care to local pregnant women, many high-risk patients are referred to our hospital, resulting in an increase in the incidence. Nevertheless, the study by Sniders et al. was a multi-center study in the United Kingdom, which was more similar to a general population. Therefore, the incidence of increased NT is closer to 5%.

As known to all, fetuses with increased NT are at high risk of chromosomal abnormalities. It has been reported that increased NT is associated with chromosomal anomalies, monogenic conditions and heart malformations, etc. (10, 13–15). However, Cut-off value for defining an increased NT is not unified in different centers. The American College of Obstetricians and Gynecologists and the Society for Maternal-Fetal Medicine (SMFM) recently recommended offering genetic counseling and diagnostic testing for enlarged NT at 3.0 mm or above the 99th centile (16). In China, 2.5 mm was used as a criteria for increase NT and as an indication for invasive procedure. Therefore, 116 fetuses (43.9%) of our study were not offered the option of invasive procedure, thereby resulting in the loss of chromosomal results.

In this study, 132 pregnant women chose invasive prenatal diagnosis. Consequently, 16 (12.1%) cases of chromosomal abnormalities were detected, the incidence of which was slightly lower than that of Kagan et al. of 19.2% (2,168/11,315). However, the incidence of chromosomal abnormalities in different subgroups were similar with Kagan et al.’s result, such as [95th centile −3.5 mm) group (5.8% vs. 7.1%), [3.5–4.5) mm group (26.7% vs. 20.1%), [4.5–5.5) mm group (42.9% vs. 45.4%). Additionally, compared to the result of Kagan et al., there was a significant difference in the [5.5–6.5 mm) group (100% vs. 50.1%) and ≥ 6.5 mm group (33.3% vs. 72.6%) (17). The reason was possibly because the number of positive cases were small, for example, there was only 1 case in [5.5–6.5 mm) group. In the ≥6.5 mm group, only 6 out of 15 cases underwent invasive diagnosis test, while the remaining 9 cases declined due to one case of IUD and eight cases of TOP. In another study using the 95th percentile as the cut-off value, Äyräs et al. revealed that the incidence of chromosomal abnormalities was 21% (7), which was similar to the results of Kagan et al. In the study by Kagan et al., the incidence of chromosomal abnormalities was as high as 39.2% (17). The authors explained that the study population was not representative of the general population, because many pregnancies with a high risk of chromosomal abnormalities would be referred to their center, resulting in an increased incidence (18).

Among the chromosomal abnormalities in this study, trisomy 21 accounted for 37.5% (6/16) of the abnormal cases. The NT thickness of trisomy 21 was mainly distributed between the 95th percentile and 4.5 mm; however, the percentage of trisomy 18 was 25% (4/16), and the NT thickness was mainly above 4.5 mm. Additionally, one case of 45, XO (6.25%) was note with the NT thickness was greater than 6.5 mm. In the study of Kagan et al., trisomy 21 was the main abnormality (54.0%, 1170/11315), followed by trisomy 18 (19.3%) and 45, XO (9.7%). More importantly, a trend was found in this study and other literature, which was that NT in trisomy 21 fetuses ranged from low to moderate thickening NT, whereas NT in trisomy 18 and 45, XO fetuses corresponds to relatively more thickening NT measurement (17, 18).

With the application of NIPT, Miranda et al. proposed the question regarding whether to recommend NIPT for fetuses with thickened NT. They found that targeted NIPT (only for trisomy 21, 18, or 13) and extended NIPT (targeted NIPT plus sex chromosomes) had missed diagnosis rates of 12 and 19% for chromosomal abnormalities, respectively. The missed diagnoses were mainly sex chromosomal abnormalities, pathogenic CNVs and Noonan syndrome (19). According to the results from Xie et al. NIPT-Plus could find 84.6% (22/26) chromosomal abnormalities (20). In this study, 2 cases were found to be at a high risk of trisomy 21 by NIPT, and the final amniocentesis results suggested that 1 case of trisomy 21, with an accurate rate of 50%. Furthermore, no false-negative cases were noted in this study. However, in this study, NIPT detection was only carried out in 80 cases, 73 of which (91.3%) had fetal NT thicknesses within 2.5 mm, and most of these fetuses did not undergo further prenatal diagnosis; thus, the results were not representational.

According to the Society for Maternal-Fetal Medicine (SMFM) guidelines, CMA has been recommended as the preferred examination method for fetal prenatal diagnosis to detect aneuploidy, fragment duplication/deletion and CNV abnormalities (21). According to Armour et al., when fetal NT was at or more than 3.5 mm, CMA should be used for prenatal diagnosis (22). However, the reported results were conflicting and variable. Huang et al. reported no known pathogenic CNV in 215 singleton pregnancies with NT more than 3.5 mm (23), while Lund reported pathogenic CNV as a rate of 12.8% (24). In our study, a sum of 4 cases of CNV abnormalities were found. There were 2 cases of abnormal CNVs discovered in the [2.5, 3.0 mm) group (4.3%, 2/47). Su et al. also suggested that one case of pathogenic and one case of likely pathogenic CNVs in the NT group 2.5–3.4 mm (1.6%) (25). Zhao et al. reported 3 (5%) cases of pathogenic CNV in the NT group of 3 and 3.5 mm (26). Furthermore, it was shown that rate of abnormal CNV in fetuses with NT from 3.1–3.4 mm was significantly higher compared to the fetuses of normal NT (27). Therefore, the cut-off for applying CMA is still under discussion.

NT thickening did not only indicate high risk of chromosomal abnormalities but it was also associated with many structural abnormalities, the most common of which was hydrops and cardiac defects. With the increasing NT thickness, the incidence of major cardiac structural abnormalities rose. When NT measurement was between 2.5 and 3.0 mm, the incidence of cardiac structural abnormalities was 2.0% (1/51). When NT measurement was more than 3.5 mm, the incidence of cardiac structural abnormalities was 15% (6/40) (28).

According to the latest study of our center, choosing the 95th percentile as the cut-off value was more sensitive than the single cut-off value of 2.5 mm[11]. It was the reason why the 95th percentile was used as the cut-off value in this study. However, in many districts in China, clinicians used 2.5 mm as a cut-off of increased NT and as an indication for invasive prenatal diagnosis (8, 20). Therefore, in this study, we further compared the percentage of chromosomal abnormalities and structural anomalies with different NT cut-offs (2.5, 3.0, 3.5 mm) (Table 4). We found that similar percentage of aneuploidy and structural defects were noted in the NT < 2.5 mm group, NT < 3.0 mm group, NT < 3.5 mm group (1.3% vs. 1.5% vs. 1.8, 6.0% vs. 7.0% vs. 8.6%).

Although there were many studies on NT thickening and chromosomal abnormalities, the feature of our study was that it truly reflected the outcomes of fetuses with increased NT in a tertiary care center in China. This study also addressed the importance to use a dynamic cut-off rather than a simple value. However, the study was not without limitations. Firstly, this was a retrospective study. Secondly, most regions in China have not customized a NT cut-off according to their own regions. The cut-off of 2.5 mm may omitted some certain occult chromosomal abnormalities. Finally, the sample size in this study was small, resulting in a relatively small number of positive cases that could be detected. In the future, our center hopes to follow up the outcomes of increased NT prospectively so as to improve counseling and management of fetuses.

## Data availability statement

The raw data supporting the conclusions of this article will be made available by the authors, without undue reservation.

## Ethics statement

This study involving human participants was reviewed and approved by the Institutional Review Board of Peking University First Hospital [protocol code 2013(572)]. The patients/participants provided their written informed consent to participate in this study.

## Author contributions

HJZ and SW designed the research and drafted the manuscript. CLF, HYZ, and WWZ performed data collection and literature search. YS and HXY were chief director of maternal and fetal unit and participated in its design and coordination. HXY provided substantial edits and additions to the manuscript. All authors have accepted responsibility for the entire content of this manuscript and approved its submission.

## Conflict of interest

The authors declare that the research was conducted in the absence of any commercial or financial relationships that could be construed as a potential conflict of interest.

## Publisher’s note

All claims expressed in this article are solely those of the authors and do not necessarily represent those of their affiliated organizations, or those of the publisher, the editors and the reviewers. Any product that may be evaluated in this article, or claim that may be made by its manufacturer, is not guaranteed or endorsed by the publisher.
